# Bis(2,2′-bipyridyl-κ^2^ *N*,*N*′)bis­(2-hydroxy­benzoato)-κ*O* ^1^;κ^2^ *O* ^1^,*O* ^1′^-cadmium(II) methanol solvate

**DOI:** 10.1107/S1600536809054610

**Published:** 2010-01-16

**Authors:** Rymel Benrabah, Bernard Viossat, Alain Tomas, Pascale Lemoine

**Affiliations:** a Laboratoire de Cristallographie et RMN Biologiques, UMR 8015 CNRS, Faculté des Sciences Pharmaceutiques et Biologiques de Paris Descartes, 4, avenue de l’Observatoire 75270 Paris Cedex 06, France

## Abstract

The title compound, [Cd(C_7_H_5_O_3_)_2_(C_10_H_8_N_2_)_2_]·CH_3_OH, contains one monomeric seven-coordinate cadmium complex and one methanol solvate mol­ecule. The Cd^II^ atom is coordinated to two 2,2′-bipyridyl ligands *via* the N atoms and to two salicylate anions (Hsal^−^) *via* the carboxyl­ate O atoms, which act as monodentate ligand for the one and bidentate ligand for the second. The Cd^II^ atom exhibits a {6 + 1} environment, approximately described as a distorted capped octa­hedron with the apical positions occupied by one of the two N atoms belonging to one bipyridyl ligand and one of the two carboxyl­ate O atoms from the monodentate Hsal^−^ ligand. Two intra­molecular six-membered hydrogen-bonded rings are present, generated from inter­actions between the carboxyl­ate and hydr­oxy groups of the salicylate ligands. There is one inter­molecular hydrogen-bonding inter­action involving the methanol solvent mol­ecule and the carboxyl­ate group from the monodentate Hsal^−^ ligand. The crystal packing is governed by π–π stacking inter­actions [centroid–centroid distance = 3.783 (4) Å] which occur between bipyridyl ligands, by C—H⋯O and C—H⋯π inter­actions and by numerous van der Waals contacts.

## Related literature

For related structures, see: Lemoine *et al.* (2004 ▶); Mazurier *et al.* (2000 ▶); Tomas *et al.* (2006 ▶); Turner *et al.* (1982 ▶). For the anti-inflammatory properties of zinc complexes, see: Sorensen (2002 ▶ and references therein).
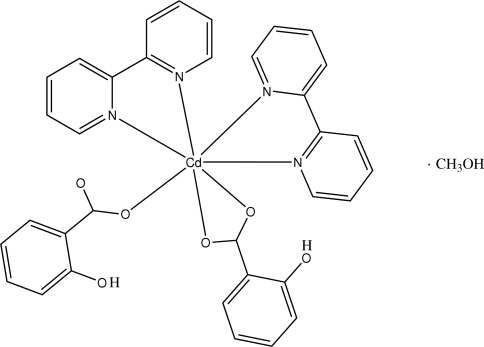


## Experimental

### 

#### Crystal data

[Cd(C_7_H_5_O_3_)_2_(C_10_H_8_N_2_)_2_]·CH_4_O
*M* *_r_* = 731.03
Triclinic, 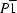

*a* = 9.115 (4) Å
*b* = 12.189 (2) Å
*c* = 14.883 (2) Å
α = 97.64 (1)°
β = 92.30 (3)°
γ = 101.00 (3)°
*V* = 1605.1 (8) Å^3^
*Z* = 2
Mo *K*α radiation
μ = 0.74 mm^−1^
*T* = 293 K
0.40 × 0.18 × 0.13 mm

#### Data collection

Enraf–Nonius CAD-4 diffractometer
9433 measured reflections
9117 independent reflections
4943 reflections with *I* > 2σ(*I*)
*R*_int_ = 0.040
3 standard reflections every 60 min  intensity decay: none

#### Refinement

*R*[*F* ^2^ > 2σ(*F* ^2^)] = 0.048
*wR*(*F* ^2^) = 0.138
*S* = 0.97
9117 reflections
423 parameters
H-atom parameters constrained
Δρ_max_ = 0.37 e Å^−3^
Δρ_min_ = −0.85 e Å^−3^

### 

Data collection: *CAD-4 EXPRESS* (Enraf–Nonius, 1994 ▶); cell refinement: *CAD-4 EXPRESS*; data reduction: *XCAD4* (Harms & Wocadlo, 1995 ▶); program(s) used to solve structure: *SIR92* (Altomare *et al.*, 1994 ▶); program(s) used to refine structure: *SHELXL97* (Sheldrick, 2008 ▶); molecular graphics: *CAMERON* (Watkin *et al.*, 1996 ▶); software used to prepare material for publication: *WinGX* (Farrugia, 1999 ▶).

## Supplementary Material

Crystal structure: contains datablocks global, I. DOI: 10.1107/S1600536809054610/dn2517sup1.cif

Structure factors: contains datablocks I. DOI: 10.1107/S1600536809054610/dn2517Isup2.hkl

Additional supplementary materials:  crystallographic information; 3D view; checkCIF report

## Figures and Tables

**Table 1:** Hydrogen-bond geometry (Å, °)

*D*—H⋯*A*	*D*—H	H⋯*A*	*D*⋯*A*	*D*—H⋯*A*
O3—H3⋯O1	0.82	1.82	2.545 (7)	147
O13—H13⋯O11	0.82	1.78	2.505 (4)	147
O61—H61⋯O12	0.82	1.98	2.802 (6)	177
C23—H23⋯O61^i^	0.93	2.57	3.429 (8)	154
C43—H43⋯O12^ii^	0.93	2.43	3.356 (6)	172
C45—H45⋯O13^iii^	0.93	2.47	3.370 (6)	163
C15—H15⋯*Cg*1^iv^	0.93	2.81	3.620 (6)	147
C47—H47⋯*Cg*2^v^	0.93	2.79	3.589 (6)	145
C62—H62*A*⋯*Cg*1^vi^	0.96	2.94	3.858 (8)	160

Symmetry codes: (i) 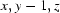; (ii) 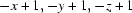; (iii) 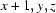; (iv) 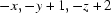; (v) 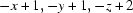; (vi) 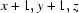. *Cg*1 and *Cg*2 are the centroids of the C2–C7 and C12–C17 rings, respectively.

**Fig. 1.**
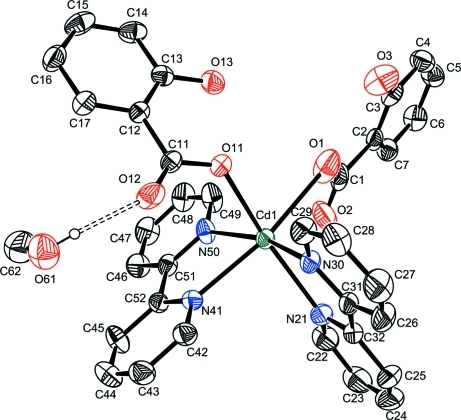
Molecular view of the solvate methanol complex showing atomic numbering and O—H–O hydrogen bonds in dotted lines. Displacements ellipsoïds are drawn at the 30% probability level.

## supplementary crystallographic information

### Comment

Recently we described the crystal structure of bis(3,5-diisopropylsalicylato)(2,9-dimethyl-1,10-phenanthroline)cadmium(II) complex (Tomas *et al.*, 2006) as an extension of an ongoing crystal structure investigation of non-steroidal anti-inflammatory drug zinc carboxylate complexes. Zinc is an essential metalloelement required by all cells for activation of a large number of Zn-dependent enzymes (Sorenson, 2002 and references therein). Various zinc carboxylates exhibit favorable anti-inflammatory, analgesic and antipyretic properties. In previous work, we have synthesized and structurally characterized two ternary complexes of Zn(II) with 3,5-diisopropylsalicylate and 1,10-phenanthroline or 2,9-dimethyl-1,10-phenanthroline (Lemoine *et al.*, 2004). Anticonvulsant and rotorod toxicity activities of these complexes were determined to examine structure-anticonvulsant and structure-hypnotic activities of these Zn(II) non- steroidal antiinflammatory agent complexes. Since zinc and cadmium belong to the same group 12 of the periodic system of elements, following this work, we report in this paper the synthesis and the structure of the bis(2,2'-bipyridyl- κ^2^*N*,*N*')(salicylato-κ^2^*O*,*O*')(salicylato-κ*O*)cadmium(II) methanol solvate.

The title compound contains one monomeric seven- coordinate cadmium complex and one methanol solvate molecule. Cd^II^ is coordinate-covalently bonded to two 2,2'-bipyridyl bidendate ligands *via* N21 and N30 or N41 and N50 atoms and to two salicylate (Hsal^-^) anionic ligands, one of which is monodentate *via* O11 atom and the other is bidentate *via* O1 and O2 atoms (Fig. 1). Therefore, the cadmium atom exhibits a {6 + 1} environment, approximately described as a distorted capped octahedron. According to this description, the Cd atom lies 0.127 (2) Å out of the basal plane [O1, N30, N41, N50] with the apical positions occupied by N21 and O11. The O2 atom caps the triangular face formed by O1, N21 and N50. This very irregular geometry is imposed by the small bite angles of one salicylate [O1—Cd—O2, 50.7 (2)°] and the two bipyridyl [N21—Cd—N30, 69.0 (1) °; N41—Cd—N50, 69.3 (1) °] bidentate ligands, mainly due to the rigidity of these chelate molecules. The Cd—N bond lengths are very similar [average 2.367 (4) Å] and comparable with distances found in other bipyridine containing Cd complexes (Turner *et al.*, 1982). The distances between Cd and the oxygen atoms are the following: d(Cd—O1) = 2.391 (5) Å and d(Cd—O2) = 2.685 (5) Å for the first salicylate ligand and d(Cd—O11) = 2.305 (3) Å for the second ligand. The longer Cd—O2 distance is comparable to that described in the bis(salicylato)(2,2'-bipyridyl) (dimethylformamide)cadmium(II) (Mazurier *et al.*, 2000) in a (6 + 1) environment (2.667 Å). The bond lengths and angles of the complexed salicylate ligands and 2,2'-bipyridyl are normal. The chelation of Cd^II^ by the Hsal^-^ ligand leads to planar ring, P1 (O1/C1/O2/Cd) with the maximum deviation of 0.026 (4) Å for C1 atom. The phenyl mean planes P2 [C2—C7] and P3 [C12—C17] [maximum deviation of 0.003 (4) Å for C7] in the salicylate ligands make a dihedral 70.5 (2) °. The bipyridyl ligands are essentially planar (maximum deviation of 0.067 (5) Å for C48) and the Cd^II^ atom is displaced from the least-squares planes P4 [N21/C22- C29/N30/C31—C32] by 0.139 (5) Å and from P5 [N41/C42- C49/N50/C51—C52] by 0.251 (5) Å. The dihedral angles between planes P4 and P5 is 82.1 (1) °.

The hydroxy H3 (or H13) atom attached to O3 (or O13) is involved in intramolecular *via* O1 (or O11) atom (Table 1) thus contributing to planarity of the rings [O1 C1 C2 C3 O3 H3 and O11 C11 C12 C13 O13 H13] (maximum deviation of 0.022 (2) Å for H3). There is one intermolecular hydrogen bonding interaction involving the solvate methanol molecule and the carboxylate O12 atom (Table 1).

Moreover, the packing is governed by π - π stacking interactions which occur between bipyridyl ligands [N21/C22—C25/C32 and N30/C26—C29/C31] through inversion centre at (1/2, 1/2, 1/2) with a centroid-to-centroid distance of 3.783 (4) Å, an average spacing of 3.50 Å with an offset of 22.1°. In addition, there are three C—H···O interactions (Table 1) and three C– H···*Cg*(π-ring) interactions (Table 1, Cg1 being the centroid of the ring C2 to C7 and Cg2 the centroid of the ring C12 to C17).

### Experimental

The cadmium(II) salicylate [Cd^II^(Sal)_2_] was prepared by reaction of Cd(NO_3_)_2_.4H_2_O with NaSal after neutralizing HSal by a NaOH solution. An amount of 0.387 g of Cd^II^(Sal)_2_ (1 mmole) and 0.313 g of 2,2'-bipyridyl (2 mmole) were dissolved in 20 ml of methanol. The solution was stirred at 60°C under reflux conditions for one hour to give the complex. Single crystals were obtained by slow evaporation of this solution at room temperature (293 K).

### Refinement

All H atoms were positioned geometrically and refined using a riding model with distances C—H = 0.96 Å (CH_3_) with *U*_iso_(H) = 1.5 times *U*_eq_(C) or 0.93 Å (CH phenyl) with *U*_iso_(H) = 1.2 times *U*_eq_(C); O—H = 0.82 Å (O3, O13 and O61) with *U*_iso_(H) = 1.2 times *U*_eq_(O).

### Crystal data

**Table:**

[Cd(C_7_H_5_O_3_)_2_(C_10_H_8_N_2_)_2_]·CH_4_O	*Z* = 2
*M**_r_* = 731.03	*F*(000) = 744
Triclinic, *P*1	*D*_x_ = 1.513 Mg m^−^^3^*D*_m_ = 1.53 (2) Mg m^−^^3^*D*_m_ measured by flotation (CCl_4_/CH_2_Cl_2_)
Hall symbol: -P 1	Mo *K*α radiation, λ = 0.71073 Å
*a* = 9.115 (4) Å	Cell parameters from 25 reflections
*b* = 12.189 (2) Å	θ = 1.7–8.9°
*c* = 14.883 (2) Å	µ = 0.74 mm^−^^1^
α = 97.64 (1)°	*T* = 293 K
β = 92.30 (3)°	Parallelepiped, colourless
γ = 101.00 (3)°	0.40 × 0.18 × 0.13 mm
*V* = 1605.1 (8) Å^3^	

### Data collection

**Table:**

Enraf–Nonius CAD-4 diffractometer	*R*_int_ = 0.040
Radiation source: fine-focus sealed tube	θ_max_ = 30.0°, θ_min_ = 2.1°
graphite	*h* = −12→12
ω–2θ scans	*k* = −17→16
9433 measured reflections	*l* = 0→20
9117 independent reflections	3 standard reflections every 60 min
4943 reflections with *I* > 2σ(*I*)	intensity decay: none

### Refinement

**Table:**

Refinement on *F*^2^	Primary atom site location: structure-invariant direct methods
Least-squares matrix: full	Secondary atom site location: difference Fourier map
*R*[*F*^2^ > 2σ(*F*^2^)] = 0.048	Hydrogen site location: inferred from neighbouring sites
*wR*(*F*^2^) = 0.138	H-atom parameters constrained
*S* = 0.97	*w* = 1/[σ^2^(*F*_o_^2^) + (0.0472*P*)^2^] where *P* = (*F*_o_^2^ + 2*F*_c_^2^)/3
9117 reflections	(Δ/σ)_max_ = 0.001
423 parameters	Δρ_max_ = 0.37 e Å^−^^3^
0 restraints	Δρ_min_ = −0.85 e Å^−^^3^

### Special details

**Table:**

Geometry. All e.s.d.'s (except the e.s.d. in the dihedral angle between two l.s. planes) are estimated using the full covariance matrix. The cell e.s.d.'s are taken into account individually in the estimation of e.s.d.'s in distances, angles and torsion angles; correlations between e.s.d.'s in cell parameters are only used when they are defined by crystal symmetry. An approximate (isotropic) treatment of cell e.s.d.'s is used for estimating e.s.d.'s involving l.s. planes.
Refinement. Refinement of *F*^2^ against ALL reflections. The weighted *R*-factor *wR* and goodness of fit *S* are based on *F*^2^, conventional *R*-factors *R* are based on *F*, with *F* set to zero for negative *F*^2^. The threshold expression of *F*^2^ > σ(*F*^2^) is used only for calculating *R*-factors(gt) *etc*. and is not relevant to the choice of reflections for refinement. *R*-factors based on *F*^2^ are statistically about twice as large as those based on *F*, and *R*- factors based on ALL data will be even larger.

### Fractional atomic coordinates and isotropic or equivalent isotropic displacement parameters (Å^2^)

**Table:**

	*x*	*y*	*z*	*U*_iso_*/*U*_eq_	
Cd1	0.22853 (3)	0.25632 (3)	0.68934 (2)	0.04219 (10)	
O1	−0.0186 (6)	0.1908 (3)	0.7336 (3)	0.1081 (18)	
O2	0.1325 (5)	0.0950 (5)	0.7918 (3)	0.1118 (18)	
O3	−0.2935 (6)	0.1625 (4)	0.7678 (3)	0.0960 (14)	
H3	−0.2179	0.1873	0.7431	0.115*	
C1	0.0072 (8)	0.1196 (5)	0.7824 (4)	0.081 (2)	
C2	−0.1228 (5)	0.0620 (4)	0.8296 (3)	0.0522 (11)	
C3	−0.2629 (6)	0.0880 (4)	0.8216 (3)	0.0570 (12)	
C4	−0.3779 (6)	0.0353 (5)	0.8688 (4)	0.0689 (14)	
H4	−0.4730	0.0519	0.8634	0.083*	
C5	−0.3499 (6)	−0.0414 (5)	0.9234 (4)	0.0707 (14)	
H5	−0.4266	−0.0764	0.9554	0.085*	
C6	−0.2113 (7)	−0.0672 (5)	0.9316 (4)	0.0716 (14)	
H6	−0.1937	−0.1193	0.9690	0.086*	
C7	−0.0989 (6)	−0.0162 (4)	0.8848 (3)	0.0617 (12)	
H7	−0.0046	−0.0344	0.8900	0.074*	
O11	0.1346 (4)	0.4193 (2)	0.7177 (2)	0.0562 (8)	
O12	0.2733 (5)	0.5535 (3)	0.6553 (2)	0.0748 (11)	
O13	0.0194 (4)	0.4604 (3)	0.8654 (2)	0.0648 (9)	
H13	0.0384	0.4223	0.8193	0.078*	
C11	0.1982 (5)	0.5224 (3)	0.7164 (3)	0.0442 (9)	
C12	0.1700 (4)	0.6045 (3)	0.7947 (3)	0.0407 (8)	
C13	0.0823 (5)	0.5701 (4)	0.8645 (3)	0.0467 (10)	
C14	0.0580 (6)	0.6490 (5)	0.9356 (3)	0.0645 (13)	
H14	−0.0014	0.6260	0.9817	0.077*	
C15	0.1213 (7)	0.7609 (5)	0.9381 (3)	0.0740 (16)	
H15	0.1056	0.8132	0.9863	0.089*	
C16	0.2082 (6)	0.7965 (4)	0.8697 (4)	0.0696 (14)	
H16	0.2503	0.8726	0.8715	0.083*	
C17	0.2322 (6)	0.7187 (4)	0.7989 (3)	0.0558 (11)	
H17	0.2911	0.7428	0.7530	0.067*	
N21	0.2855 (4)	0.0894 (3)	0.6089 (3)	0.0526 (9)	
C22	0.3627 (6)	0.0235 (5)	0.6465 (4)	0.0751 (15)	
H22	0.3981	0.0448	0.7071	0.090*	
C23	0.3936 (7)	−0.0744 (5)	0.6013 (6)	0.090 (2)	
H23	0.4506	−0.1174	0.6294	0.108*	
C24	0.3379 (8)	−0.1058 (5)	0.5145 (6)	0.098 (2)	
H24	0.3548	−0.1727	0.4824	0.118*	
C27	−0.0004 (7)	0.1813 (6)	0.3642 (4)	0.0818 (17)	
H27	−0.0448	0.1652	0.3053	0.098*	
C28	−0.0146 (6)	0.2783 (5)	0.4192 (4)	0.0712 (15)	
H28	−0.0696	0.3282	0.3991	0.085*	
C29	0.0553 (6)	0.2984 (4)	0.5045 (3)	0.0605 (12)	
H29	0.0483	0.3645	0.5419	0.073*	
N30	0.1333 (4)	0.2278 (3)	0.5370 (2)	0.0472 (8)	
C31	0.1458 (5)	0.1334 (4)	0.4834 (3)	0.0496 (10)	
C26	0.0791 (6)	0.1089 (5)	0.3964 (3)	0.0727 (15)	
H26	0.0884	0.0430	0.3596	0.087*	
C25	0.2571 (7)	−0.0412 (4)	0.4727 (4)	0.0771 (16)	
H25	0.2196	−0.0629	0.4126	0.092*	
C32	0.2323 (5)	0.0582 (4)	0.5224 (3)	0.0512 (10)	
N41	0.4715 (4)	0.3495 (3)	0.6612 (2)	0.0459 (8)	
C42	0.5095 (5)	0.3697 (4)	0.5786 (3)	0.0547 (11)	
H42	0.4367	0.3481	0.5306	0.066*	
C43	0.6514 (6)	0.4210 (4)	0.5607 (4)	0.0664 (13)	
H43	0.6740	0.4356	0.5025	0.080*	
C44	0.7574 (6)	0.4497 (5)	0.6315 (4)	0.0779 (16)	
H44	0.8550	0.4831	0.6219	0.094*	
C47	0.5660 (7)	0.3693 (5)	0.9829 (4)	0.0762 (16)	
H47	0.6298	0.3856	1.0353	0.091*	
C48	0.4174 (7)	0.3288 (5)	0.9872 (3)	0.0740 (16)	
H48	0.3772	0.3186	1.0427	0.089*	
C49	0.3281 (7)	0.3034 (5)	0.9085 (3)	0.0741 (16)	
H49	0.2265	0.2752	0.9118	0.089*	
N50	0.3790 (4)	0.3170 (3)	0.8270 (2)	0.0545 (9)	
C51	0.5237 (5)	0.3598 (4)	0.8221 (3)	0.0482 (10)	
C46	0.6214 (6)	0.3861 (5)	0.8992 (3)	0.0665 (13)	
H46	0.7227	0.4146	0.8949	0.080*	
C45	0.7201 (5)	0.4292 (5)	0.7171 (4)	0.0704 (14)	
H45	0.7922	0.4481	0.7655	0.084*	
C52	0.5742 (4)	0.3801 (3)	0.7306 (3)	0.0447 (9)	
O61	0.5469 (6)	0.7016 (4)	0.6474 (3)	0.1025 (14)	
H61	0.4652	0.6606	0.6503	0.123*	
C62	0.6266 (9)	0.7191 (6)	0.7309 (5)	0.110 (2)	
H62A	0.6469	0.7982	0.7543	0.165*	
H62B	0.7195	0.6937	0.7239	0.165*	
H62C	0.5689	0.6777	0.7724	0.165*	

### Atomic displacement parameters (Å^2^)

**Table:**

	*U*^11^	*U*^22^	*U*^33^	*U*^12^	*U*^13^	*U*^23^
Cd1	0.04206 (16)	0.04538 (17)	0.03578 (14)	0.00416 (11)	−0.00069 (11)	0.00118 (11)
O1	0.169 (5)	0.061 (2)	0.080 (3)	−0.021 (3)	0.061 (3)	0.007 (2)
O2	0.071 (3)	0.145 (5)	0.090 (3)	−0.034 (3)	0.022 (3)	−0.014 (3)
O3	0.140 (4)	0.085 (3)	0.078 (3)	0.044 (3)	0.019 (3)	0.031 (2)
C1	0.095 (5)	0.069 (4)	0.054 (3)	−0.033 (3)	0.029 (3)	−0.022 (3)
C2	0.061 (3)	0.045 (2)	0.039 (2)	−0.011 (2)	0.014 (2)	−0.0069 (18)
C3	0.081 (3)	0.044 (2)	0.044 (2)	0.011 (2)	0.006 (2)	−0.0001 (19)
C4	0.055 (3)	0.085 (4)	0.065 (3)	0.013 (3)	0.010 (2)	0.006 (3)
C5	0.064 (3)	0.081 (4)	0.062 (3)	−0.003 (3)	0.021 (3)	0.015 (3)
C6	0.082 (4)	0.068 (3)	0.064 (3)	0.008 (3)	0.004 (3)	0.019 (3)
C7	0.058 (3)	0.067 (3)	0.056 (3)	0.007 (2)	0.000 (2)	0.002 (2)
O11	0.0567 (19)	0.0455 (17)	0.0627 (19)	0.0080 (14)	0.0092 (15)	−0.0037 (14)
O12	0.111 (3)	0.062 (2)	0.052 (2)	0.015 (2)	0.034 (2)	0.0050 (16)
O13	0.061 (2)	0.069 (2)	0.061 (2)	0.0034 (17)	0.0169 (16)	0.0076 (17)
C11	0.052 (2)	0.043 (2)	0.037 (2)	0.0111 (18)	−0.0021 (18)	0.0018 (17)
C12	0.044 (2)	0.042 (2)	0.0356 (19)	0.0112 (17)	−0.0067 (16)	0.0019 (16)
C13	0.040 (2)	0.058 (3)	0.042 (2)	0.0124 (19)	−0.0037 (17)	0.0044 (19)
C14	0.068 (3)	0.086 (4)	0.042 (2)	0.028 (3)	0.009 (2)	−0.001 (2)
C15	0.096 (4)	0.080 (4)	0.048 (3)	0.041 (3)	−0.003 (3)	−0.016 (3)
C16	0.076 (4)	0.050 (3)	0.076 (3)	0.012 (2)	−0.006 (3)	−0.012 (2)
C17	0.066 (3)	0.048 (3)	0.053 (3)	0.013 (2)	0.004 (2)	0.004 (2)
N21	0.052 (2)	0.048 (2)	0.059 (2)	0.0140 (17)	0.0044 (18)	0.0080 (17)
C22	0.067 (3)	0.071 (4)	0.094 (4)	0.019 (3)	0.012 (3)	0.027 (3)
C23	0.073 (4)	0.065 (4)	0.148 (7)	0.029 (3)	0.028 (4)	0.041 (4)
C24	0.090 (5)	0.055 (3)	0.154 (7)	0.026 (3)	0.048 (5)	0.001 (4)
C27	0.080 (4)	0.109 (5)	0.047 (3)	0.007 (4)	−0.018 (3)	0.001 (3)
C28	0.067 (3)	0.087 (4)	0.059 (3)	0.007 (3)	−0.012 (3)	0.024 (3)
C29	0.065 (3)	0.064 (3)	0.052 (3)	0.016 (2)	−0.009 (2)	0.006 (2)
N30	0.048 (2)	0.049 (2)	0.0397 (18)	0.0045 (16)	−0.0023 (15)	−0.0026 (15)
C31	0.047 (2)	0.051 (2)	0.042 (2)	−0.0027 (19)	0.0040 (18)	−0.0054 (18)
C26	0.080 (4)	0.079 (4)	0.044 (3)	0.000 (3)	−0.008 (2)	−0.017 (2)
C25	0.083 (4)	0.055 (3)	0.086 (4)	0.011 (3)	0.022 (3)	−0.015 (3)
C32	0.045 (2)	0.045 (2)	0.059 (3)	0.0016 (18)	0.009 (2)	−0.003 (2)
N41	0.0443 (19)	0.048 (2)	0.0446 (19)	0.0089 (15)	0.0057 (15)	0.0040 (15)
C42	0.057 (3)	0.061 (3)	0.047 (2)	0.010 (2)	0.008 (2)	0.008 (2)
C43	0.070 (3)	0.065 (3)	0.068 (3)	0.015 (3)	0.021 (3)	0.018 (3)
C44	0.053 (3)	0.092 (4)	0.091 (4)	0.006 (3)	0.021 (3)	0.027 (3)
C47	0.081 (4)	0.089 (4)	0.052 (3)	0.011 (3)	−0.028 (3)	0.003 (3)
C48	0.100 (4)	0.075 (4)	0.041 (3)	0.000 (3)	−0.007 (3)	0.015 (2)
C49	0.078 (4)	0.086 (4)	0.044 (3)	−0.022 (3)	−0.001 (2)	0.013 (3)
N50	0.057 (2)	0.060 (2)	0.0382 (18)	−0.0092 (18)	−0.0053 (16)	0.0098 (17)
C51	0.046 (2)	0.045 (2)	0.051 (2)	0.0090 (18)	−0.0109 (19)	0.0029 (19)
C46	0.056 (3)	0.084 (4)	0.054 (3)	0.012 (3)	−0.013 (2)	−0.001 (3)
C45	0.041 (3)	0.086 (4)	0.079 (4)	−0.005 (2)	−0.005 (2)	0.021 (3)
C52	0.037 (2)	0.044 (2)	0.053 (2)	0.0092 (17)	0.0001 (18)	0.0048 (18)
O61	0.115 (4)	0.105 (4)	0.080 (3)	−0.002 (3)	0.014 (3)	0.020 (3)
C62	0.102 (5)	0.113 (6)	0.110 (6)	0.021 (5)	0.004 (5)	0.001 (5)

### Geometric parameters (Å, °)

**Table:**

Cd1—O11	2.305 (3)	C23—H23	0.9300
Cd1—N30	2.352 (3)	C24—C25	1.365 (9)
Cd1—N50	2.368 (4)	C24—H24	0.9300
Cd1—N41	2.369 (4)	C27—C26	1.365 (8)
Cd1—N21	2.377 (4)	C27—C28	1.375 (8)
Cd1—O1	2.391 (5)	C27—H27	0.9300
Cd1—O2	2.685 (5)	C28—C29	1.368 (7)
O1—C1	1.253 (8)	C28—H28	0.9300
O2—C1	1.242 (8)	C29—N30	1.340 (6)
O3—C3	1.348 (6)	C29—H29	0.9300
O3—H3	0.8200	N30—C31	1.336 (5)
C1—C2	1.507 (7)	C31—C26	1.378 (6)
C2—C3	1.377 (7)	C31—C32	1.476 (6)
C2—C7	1.380 (7)	C26—H26	0.9300
C3—C4	1.389 (7)	C25—C32	1.394 (6)
C4—C5	1.369 (7)	C25—H25	0.9300
C4—H4	0.9300	N41—C52	1.330 (5)
C5—C6	1.364 (8)	N41—C42	1.333 (5)
C5—H5	0.9300	C42—C43	1.378 (7)
C6—C7	1.360 (7)	C42—H42	0.9300
C6—H6	0.9300	C43—C44	1.363 (8)
C7—H7	0.9300	C43—H43	0.9300
O11—C11	1.283 (5)	C44—C45	1.374 (8)
O12—C11	1.217 (5)	C44—H44	0.9300
O13—C13	1.353 (5)	C47—C48	1.358 (8)
O13—H13	0.8200	C47—C46	1.388 (7)
C11—C12	1.497 (5)	C47—H47	0.9300
C12—C17	1.391 (6)	C48—C49	1.364 (7)
C12—C13	1.395 (6)	C48—H48	0.9300
C13—C14	1.387 (6)	C49—N50	1.335 (6)
C14—C15	1.371 (8)	C49—H49	0.9300
C14—H14	0.9300	N50—C51	1.332 (6)
C15—C16	1.379 (8)	C51—C46	1.387 (6)
C15—H15	0.9300	C51—C52	1.492 (6)
C16—C17	1.375 (6)	C46—H46	0.9300
C16—H16	0.9300	C45—C52	1.384 (6)
C17—H17	0.9300	C45—H45	0.9300
N21—C22	1.326 (6)	O61—C62	1.384 (8)
N21—C32	1.337 (6)	O61—H61	0.8200
C22—C23	1.374 (8)	C62—H62A	0.9600
C22—H22	0.9300	C62—H62B	0.9600
C23—C24	1.347 (10)	C62—H62C	0.9600
			
O11—Cd1—N30	90.83 (12)	C24—C23—C22	117.4 (6)
O11—Cd1—N50	87.64 (13)	C24—C23—H23	121.3
N30—Cd1—N50	164.41 (12)	C22—C23—H23	121.3
O11—Cd1—N41	95.01 (12)	C23—C24—C25	121.1 (6)
N30—Cd1—N41	95.40 (12)	C23—C24—H24	119.5
N50—Cd1—N41	69.31 (12)	C25—C24—H24	119.5
O11—Cd1—N21	159.80 (13)	C26—C27—C28	119.7 (5)
N30—Cd1—N21	68.98 (13)	C26—C27—H27	120.2
N50—Cd1—N21	111.80 (14)	C28—C27—H27	120.2
N41—Cd1—N21	87.26 (13)	C29—C28—C27	117.5 (5)
O11—Cd1—O1	76.61 (14)	C29—C28—H28	121.2
N30—Cd1—O1	89.34 (15)	C27—C28—H28	121.2
N50—Cd1—O1	105.36 (15)	N30—C29—C28	123.3 (5)
N41—Cd1—O1	170.46 (12)	N30—C29—H29	118.4
N21—Cd1—O1	102.18 (14)	C28—C29—H29	118.4
O11—Cd1—O2	116.15 (14)	C31—N30—C29	118.9 (4)
N30—Cd1—O2	117.82 (13)	C31—N30—Cd1	119.0 (3)
N50—Cd1—O2	76.51 (13)	C29—N30—Cd1	121.8 (3)
N41—Cd1—O2	132.29 (14)	N30—C31—C26	120.6 (5)
N21—Cd1—O2	75.45 (14)	N30—C31—C32	116.8 (4)
O1—Cd1—O2	50.73 (17)	C26—C31—C32	122.6 (4)
C1—O1—Cd1	100.0 (5)	C27—C26—C31	120.1 (5)
C1—O2—Cd1	86.3 (4)	C27—C26—H26	120.0
C3—O3—H3	109.5	C31—C26—H26	120.0
O2—C1—O1	122.8 (6)	C24—C25—C32	118.4 (6)
O2—C1—C2	120.3 (7)	C24—C25—H25	120.8
O1—C1—C2	116.9 (7)	C32—C25—H25	120.8
C3—C2—C7	119.3 (4)	N21—C32—C25	120.9 (5)
C3—C2—C1	121.9 (5)	N21—C32—C31	116.6 (4)
C7—C2—C1	118.7 (5)	C25—C32—C31	122.4 (5)
O3—C3—C2	122.0 (5)	C52—N41—C42	119.2 (4)
O3—C3—C4	118.2 (5)	C52—N41—Cd1	118.3 (3)
C2—C3—C4	119.8 (5)	C42—N41—Cd1	122.5 (3)
C5—C4—C3	119.3 (5)	N41—C42—C43	123.2 (5)
C5—C4—H4	120.3	N41—C42—H42	118.4
C3—C4—H4	120.3	C43—C42—H42	118.4
C6—C5—C4	121.0 (5)	C44—C43—C42	117.5 (5)
C6—C5—H5	119.5	C44—C43—H43	121.2
C4—C5—H5	119.5	C42—C43—H43	121.2
C7—C6—C5	119.6 (5)	C43—C44—C45	120.0 (5)
C7—C6—H6	120.2	C43—C44—H44	120.0
C5—C6—H6	120.2	C45—C44—H44	120.0
C6—C7—C2	120.9 (5)	C48—C47—C46	119.1 (5)
C6—C7—H7	119.5	C48—C47—H47	120.4
C2—C7—H7	119.5	C46—C47—H47	120.4
C11—O11—Cd1	130.0 (3)	C47—C48—C49	118.6 (5)
C13—O13—H13	109.5	C47—C48—H48	120.7
O12—C11—O11	123.5 (4)	C49—C48—H48	120.7
O12—C11—C12	121.3 (4)	N50—C49—C48	123.5 (5)
O11—C11—C12	115.2 (4)	N50—C49—H49	118.3
C17—C12—C13	118.5 (4)	C48—C49—H49	118.3
C17—C12—C11	119.7 (4)	C51—N50—C49	118.5 (4)
C13—C12—C11	121.9 (4)	C51—N50—Cd1	118.1 (3)
O13—C13—C14	118.1 (4)	C49—N50—Cd1	123.3 (3)
O13—C13—C12	121.7 (4)	N50—C51—C46	121.1 (4)
C14—C13—C12	120.1 (4)	N50—C51—C52	116.9 (4)
C15—C14—C13	120.0 (5)	C46—C51—C52	122.0 (4)
C15—C14—H14	120.0	C47—C46—C51	119.1 (5)
C13—C14—H14	120.0	C47—C46—H46	120.5
C14—C15—C16	120.7 (4)	C51—C46—H46	120.5
C14—C15—H15	119.7	C44—C45—C52	119.4 (5)
C16—C15—H15	119.7	C44—C45—H45	120.3
C17—C16—C15	119.5 (5)	C52—C45—H45	120.3
C17—C16—H16	120.3	N41—C52—C45	120.7 (4)
C15—C16—H16	120.3	N41—C52—C51	116.8 (4)
C16—C17—C12	121.2 (5)	C45—C52—C51	122.5 (4)
C16—C17—H17	119.4	C62—O61—H61	109.5
C12—C17—H17	119.4	O61—C62—H62A	109.5
C22—N21—C32	118.4 (4)	O61—C62—H62B	109.5
C22—N21—Cd1	123.3 (4)	H62A—C62—H62B	109.5
C32—N21—Cd1	118.3 (3)	O61—C62—H62C	109.5
N21—C22—C23	123.7 (6)	H62A—C62—H62C	109.5
N21—C22—H22	118.1	H62B—C62—H62C	109.5
C23—C22—H22	118.1		
			
O2—C1—C2—C3	179.3 (5)	Cd1—O12—C11—C12	158.7 (5)
O1—C1—C2—C3	−1.5 (7)	Cd1—O11—C11—C12	−135.3 (3)
O2—C1—C2—C7	1.5 (7)	C11—C12—C13—O13	0.7 (6)
O1—C1—C2—C7	−179.4 (4)	Cd1—N30—C29—C28	−173.1 (4)
Cd1—O2—C1—C2	−177.0 (4)	Cd1—N30—C31—C32	−5.6 (5)
Cd1—O1—C1—C2	176.4 (3)	Cd1—N30—C31—C26	173.9 (4)
C1—C2—C3—O3	3.1 (7)	Cd1—N21—C22—C23	178.6 (4)
O12—C11—C12—C13	179.0 (4)	Cd1—N21—C32—C31	1.9 (5)
O11—C11—C12—C13	0.9 (6)	Cd1—N50—C51—C52	8.0 (5)
O12—C11—C12—C17	−1.0 (6)	Cd1—N21—C32—C25	−177.6 (4)
O11—C11—C12—C17	−179.1 (4)		

### Hydrogen-bond geometry (Å, °)

**Table:**

Cg1 and Cg2 are the centroids of the C2–C7 and C12–C17 rings, respectively.

**Table:**

*D*—H···*A*	*D*—H	H···*A*	*D*···*A*	*D*—H···*A*
O3—H3···O1	0.82	1.82	2.545 (7)	147
O13—H13···O11	0.82	1.78	2.505 (4)	147
O61—H61···O12	0.82	1.98	2.802 (6)	177
C23—H23···O61^i^	0.93	2.57	3.429 (8)	154
C43—H43···O12^ii^	0.93	2.43	3.356 (6)	172
C45—H45···O13^iii^	0.93	2.47	3.370 (6)	163
C15—H15···Cg1^iv^	0.93	2.81	3.620 (6)	147
C47—H47···Cg2^v^	0.93	2.79	3.589 (6)	145
C62—H62A···Cg1^vi^	0.96	2.94	3.858 (8)	160

Symmetry codes: (i) *x*, *y*−1, *z*; (ii) −*x*+1, −*y*+1, −*z*+1; (iii) *x*+1, *y*, *z*; (iv) −*x*, −*y*+1, −*z*+2; (v) −*x*+1, −*y*+1, −*z*+2; (vi) *x*+1, *y*+1, *z*.
